# Tribute to Dr Raymond Downing: 1949–2020

**DOI:** 10.4102/phcfm.v12i1.2386

**Published:** 2020-04-07

**Authors:** Jeremiah Laktabai

**Affiliations:** 1 Department of Family Medicine, College of Health Sciences, Moi University, Eldoret, Kenya

Raymond Victor Downing was born on 16 February 1949 in Massachusetts, USA. He was the second of three children born to Harry and Ebba Downing. He was brought up in the Plymouth Brethren church in Auburn, Massachusetts, and lived in Massachusetts for his early childhood. In 1967, he went to Tufts University, and in 1975, received his Doctor of Medicine (MD) from New York Medical College. Afterwards, Raymond completed his residency in Family Medicine at the University of Tennessee Hospital and worked in Tennessee for 7 years. He met Dr Janice Armstrong, also a family physician, during his stay in Tennessee and they married in October 1977. There they had two children: Elizabeth and Timothy.

In 1985, they moved with their two young children to Sudan to work in a refugee settlement clinic. From there, in 1989, they moved to Tanzania to work in a Mennonite Mission Hospital. Four years later in 1994, they moved again to Kenya, and began working at Friends Lugulu Hospital in western Kenya. In 2005, Raymond and Janice joined the Department of Family Medicine, Moi University in Webuye, Bungoma County., and worked with other colleagues to design the first family medicine training programme in the country. In 2016, Raymond retired from Moi University, and shortly afterwards joined the medical team at Dreamland Mission Hospital, where he worked until his death.

Raymond was also a prolific writer and has written several books and essays on global health and medicine, which include his thoughts on death and dying. His most recent titles include *Such a Time of It They Had* and *Global Health Means Listening*. He was a devout Christian, a family medicine physician, mentor, author, teacher, brother, father, husband, colleague and a great friend.

Raymond passed away comfortably on Monday, 20 January 2020, in the evening at his home in Kimili, western Kenya surrounded by his entire family. He leaves behind his wife, Janice Armstrong; two sisters, Mary-Ellen Welsher and Nancy Kline; and two children, Elizabeth and Timothy Downing. He would certainly be missed.

